# Genome editing of polyploid crops: prospects, achievements and bottlenecks

**DOI:** 10.1007/s11248-021-00251-0

**Published:** 2021-04-12

**Authors:** Jan G. Schaart, Clemens C. M. van de Wiel, Marinus J. M. Smulders

**Affiliations:** Plant Breeding, Wageningen University and Research, Wageningen, Netherlands

**Keywords:** Genome editing, Polyploid crops, CRISPR/Cas, TALENs

## Abstract

Plant breeding aims to develop improved crop varieties. Many crops have a polyploid and often highly heterozygous genome, which may make breeding of polyploid crops a real challenge. The efficiency of traditional breeding based on crossing and selection has been improved by using marker-assisted selection (MAS), and MAS is also being applied in polyploid crops, which helps e.g. for introgression breeding. However, methods such as random mutation breeding are difficult to apply in polyploid crops because there are multiple homoeologous copies (alleles) of each gene. Genome editing technology has revolutionized mutagenesis as it enables precisely selecting targets. The genome editing tool CRISPR/Cas is especially valuable for targeted mutagenesis in polyploids, as all alleles and/or copies of a gene can be targeted at once. Even multiple genes, each with multiple alleles, may be targeted simultaneously. In addition to targeted mutagenesis, targeted replacement of undesirable alleles by desired ones may become a promising application of genome editing for the improvement of polyploid crops, in the near future. Several examples of the application of genome editing for targeted mutagenesis are described here for a range of polyploid crops, and achievements and bottlenecks are highlighted.

## Significance statement

Genome editing tools are evolving rapidly. This review evaluates various technologies for application of genome editing in polyploid crop species, including recent applications to heritable chromosomal rearrangements, in terms of prospects and applicability. While this article summarizes several examples of genome editing for targeted mutagenesis in polyploids, it also discusses the bottlenecks and challenges that remain to be addressed.

**Amit Dhingra,** *Department of Horticulture, Washington State University, Pullman, WA USA*

## Introduction

Many important crops that are grown for food, feed or for their biobased or ornamental value are polyploid species, meaning that their genomes have more than two homologous sets of chromosomes. Polyploid species may originate from whole genome duplication events in their evolutionary history leading to autopolyploids, which have genomes with multiple sets of homologous chromosomes. In many polyploid species the genome duplication was preceded by a hybridization of two closely related species, or the hybridization took place between unreduced or reconstituted (2n) gametes. This results in allopolyploids, which have multiple sets of homoeologous chromosomes. The distinction between autopolyploids and allopolyploids is an oversimplification, as in many cases the evolutionary history is more complex, and polyploids may have complex chromosome pairing behavior in the meiosis. For instance, Bourke et al. (2017) found disomic as well as tetrasomic behaviour in one parent of a cross in tetraploid rose, sometimes on the same chromosome, but for different chromosomes in each parent. However, the distinction is relevant as on average the sequence differences between alleles will be larger for those on homoeologous chromosomes compared to homologous chromosomes.

Breeding by crossing and selection is the essence of crop improvement, for example for introducing disease resistance traits. Polyploids have multiple alleles associated with a single locus, and as an immediate consequence, segregation is more complex when compared to diploids. Moreover, polyploid crops are often highly heterozygous (and to maintain the genetic makeup of a variety, they are therefore vegetatively propagated). The heterozygous nature makes traditional breeding of polyploid crops challenging, although application of methods such as marker-assisted selection may greatly improve breeding efficiency. Through introgression breeding, novel genetic variation from wild relatives can be introduced in crops for the improvement of important traits, in particular tolerance to biotic and abiotic stresses. Introgression of novel traits of interest is achieved by hybridization and repeated backcrossing and selection. Especially in heterozygous polyploids it takes several rounds of (pseudo)backcrossing to recover most of the crop’s original genome composition, which is laborious and often difficult to apply because of inbreeding depression.

Traditional mutation breeding using random mutations is being applied in some polyploid crops, e.g. in tetraploid or hexaploid wheat (Krasileva et al. 2017). In autopolyploid species also multiple rounds of backcrossing are required, first to get the intended mutation in homozygous state (as desired mutations are mostly recessive) and subsequently for the removal of unwanted mutations. For a full knockout mutation of a gene function in an allopolyploid species, mutations of each of the homoeologs are found in separate plant lines and must subsequently be combined by a series of crossings. To avoid these time-consuming breeding steps, alternative methods for introgression breeding and mutation breeding are important for increasing genetic variation in polyploid crops species. Genome editing is such an alternative and its value for polyploid crop improvement is discussed in this review.

## Genome editing and polyploid crops

In genome editing, also referred to as gene editing, site-specific nucleases (SSNs) such as Zinc Finger Nucleases (ZFNs), Transcription Activator-Like Effector Nucleases (TALENs) or Clustered Regularly Interspaced Short Palindromic Repeats (CRISPR)/associated (Cas) proteins are employed for inducing changes at specific genomic loci. SSNs introduce targeted double-strand breaks (DSBs) in DNA, which are repaired by the endogenous DNA repair machinery. The main repair pathways of DSBs are nonhomologous end joining (NHEJ), in which the broken DNA ends are ligated together, and homology-directed repair (HDR), which makes use of a homologous DNA repair template. In somatic plant cells, NHEJ is the commonly used DNA repair pathway. It may occasionally result in small insertion-deletion mutations (indels) at the repaired site (Charbonnel et al. 2011; Gorbunova and Levy 1997; Lloyd et al. 2012). Repair by HDR is much more precise. It may also be employed to generate specific sequence changes or sequence replacements (Puchta et al. 1996; Puchta and Fauser 2013; Rouet et al. 1994).

Because of its flexibility and high efficiency in creating targeted modifications, CRISPR/Cas is the main SSN used in contemporary genome editing studies. CRISPR/Cas consist of two components, a Cas nuclease and a guide RNA. The guide RNA is a specific RNA sequence which can be designed to recognize a target DNA sequence of interest and so to direct the Cas nuclease to this sequence for editing. Several CRISPR/Cas variants with unique properties have been isolated from different bacterial taxa. Also a diversity of modified Cas-enzymes has been engineered for genome editing, including Cas-nickases, which produce targeted single-strands nicks, base-editors, which enable targeted substitution of nucleotides, and prime-editors, which enable re-writing short stretches of genomic DNA (Anzalone et al. 2020). It is not a surprise that this rapid development and application of CRISPR/Cas has caused a revolution in the field of genetic and genomic studies.

CRISPR/Cas technology also appears to be very promising in facilitating crop breeding. Despite the wide variety of possible applications using CRISPR/Cas that have begun to be implemented in plants, presently in most studies CRISPR/Cas has been used to create targeted mutations, often small deletions, commonly to generate loss-of-function mutant alleles. Such CRISPR/Cas-mediated gene edited plants have been obtained for a variety of traits, including increased yield and growth characteristics, improved food and feed quality, increased resistance to biotic and abiotic stresses, herbicide tolerance and better industrial utilization (reviewed/inventoried in Modrzejewski et al. (2019)). Concerning targeting the genomes of polyploid crops and other crops with complex genomes, CRISPR/Cas is versatile as it has the ability to achieve simultaneous mutations at multiple genomic sites. The guide RNAs need to be designed with care so that they can target all different alleles that are to be mutated. It should however be noted that, because of the presence of multiple alleles of target genes present, establishing a CRISPR system in polyploid crops is still more challenging than in diploid crops.

In genome editing studies usually first transgenic lines are created in which the SSN genes are stably integrated into the host genome. In a subsequent step mutant plants are selected from these transgenic lines and further characterized. The SSN transgenes may finally be removed from selected mutant lines by segregation by selfing or backcrossing. In polyploid crops however backcrossing is often not an option, which is especially the case for vegetatively propagated, highly heterozygous crops. Having methods available that avoid integration of the DNA containing the SSN in the genome, and which do produce targeted mutations is important to generate non-transgenic mutant plant lines.

In this review, we describe various examples of genome editing in polyploid crops, illustrating the versatility of applications aiming at direct improvement of a range of traits.

## Eliminating genomic viral sequences and enhancing levels of β-carotene in triploid banana

Banana (*Musa* spp.) is an important staple food crop in tropical and subtropical countries. Many banana varieties are grown for local and international markets, but diseases and pests are a major factor limiting yield in commercial production. Especially the Cavendish type of dessert bananas is grown at large scale as monoculture, making it particularly susceptible to diseases for which it has no natural resistance. Conventional breeding for improvement of important commercial banana varieties is hampered by the low genetic variability in *Musa* germplasm, their triploid genomic nature causing sterility, and banana’s lengthy generation cycle (Silva et al. 2001). Application of new plant breeding techniques such as genome editing may be promising for improvement of existing triploid elite banana varieties. Kaur et al. (2018) and Naim et al. (2018) established a CRISPR/Cas protocol for triploid banana, making use of *Agrobacterium*-mediated transformation of somatic embryos for the introduction of CRISPR/Cas reagents in banana. Tripathi et al. (2019b) and Ntui et al. (2020) demonstrated efficient genome editing of triploid banana varieties, including ‘Rasthali’ (AAB-genome) and ‘Cavendish’ (AAA-genome) using CRISPR/Cas targeting the phytoene desaturase (*PDS*) gene, a key enzyme in the carotene biosynthesis pathway. Knockout of *PDS* also leads to impaired chlorophyll biosynthesis and is often used as a model system in genome editing studies for its easily recognizable (albino) phenotype. The studies reported high editing efficiency (up to 100%) of the *PDS* gene and recovered banana regenerants showing an albino phenotype, indicating full knockout of the *PDS* gene function. The albino phenotype also implies that these plants were derived from cells in which the three alleles have been edited simultaneously. This quality for multiallelic editing makes CRISPR/Cas-based genome editing a useful tool for improvement of commercially important triploid banana cultivars.

An interesting application of genome editing in banana was described by Tripathi et al. (2019a) who used CRISPR/Cas9 to target the endogenous banana streak virus (eBSV), which is integrated in the B genome of plantain. Plantains are triploid *Musa* types with an AAB-genome and originate from hybridization of *M. balbisiana* (B genome) and *M. acuminata* (A genome). Multiple copies of eBSV viral sequences have become integrated as direct and inverted tandem repeats at a single locus on chromosome 1 of the B genome (Chabannes et al. 2013). When banana plants with B-genomes come under stress, for example by drought, tissue culture or hybridization, the eBSV recombines to create a functional viral genome producing infectious viral particles, and as a result the plant develops disease symptoms. This hampers the use of *M. balbisiana* (with B genome) in breeding. Using CRISPR/Cas9 targeting the integrated sequences of eBSV in the plantain variety Gonja Manjaya (AAB genome) at three different sites, 17 out of 20 lines with a transgenic event showed mutations at the targeted locus. Most of the edited lines showed mutations at all three target sites. Eight gene-edited lines were tested in a greenhouse together with three non-edited control lines for development of BSV symptoms under water stress conditions. Six of the edited lines remained asymptomatic, as compared to the control lines, confirming inhibition of eBSV in these lines. By permanently inactivating the endogenous eBSV, banana germplasm with B genome(s) can now be used as germplasm in breeding programs for improving plantain varieties.

Recently a precise genome editing experiment to improve a nutritional trait in banana was described, in which CRISPR/Cas9 was used to develop a β-carotene-enriched Cavendish banana variety (Kaur et al. 2020). The target was the lycopene epsilon-cyclase (*LCYε*) gene. Loss of LCYε function would result in a redirection of the carotenoid biosynthetic pathway, leading to enhanced levels of β-carotene, at the expense of α-carotene and lutein biosynthesis. Several transgenic lines were produced by *Agrobacterium*-mediated transformation starting with embryogenic cell suspensions. Ten out of 12 transgenic lines that survived the transfer to the greenhouse showed indels in their *LCYε* gene sequence. Although no clear molecular analysis of the *LCYε* locus was presented, a T7 endonuclease assay indicated a homozygous deletion in a single edited line and heterozygous mutations in three other lines. These four lines were all used for further evaluation and an analysis of fruit pulp showed an up to sixfold enhanced β-carotene content compared with the unedited plants, and a complete absence or a drastic reduction of the levels of lutein and α-carotene.

## Herbicide tolerance, and improved product quality in tetraploid potato

Potato (*Solanum tuberosum*) is another important staple crop with excellent yield potential and high nutritional value (Barrell et al. 2013). Potato originates from South America and is presently grown globally, originally mostly in Europe, North America and in countries of the former Soviet Union, but nowadays China and India are the major potato producers (https://www.potatopro.com/world/potato-statistics). Most commercial potato varieties are autotetraploid, meaning that each chromosome is present as four homologous copies that pair randomly in meiosis. Breeding of improved cultivars is hampered by the high level of heterozygosity and the tetrasomic inheritance (Uitdewilligen et al. 2013). Commercial potato varieties are not propagated by seeds, but by vegetative parts, mostly tubers.

In addition to traditional breeding, quite some effort has been put in the application of genome editing for the improvement of tetraploid potato. Early work described successful editing of the acetolactate synthase (ALS), which has a potential function in herbicide resistance (Endo and Toki 2013) but which may also be employed as a selectable marker gene. Nicolia et al. (2015) and Butler et al. (2015) aimed at targeted mutagenesis of *ALS* using TALENs which were transiently expressed from plasmid DNA in potato protoplasts or using CRISPR/Cas in stably transformed potato lines, respectively. Both studies indicated an effective targeted mutagenesis of *ALS*, but no full knockout mutants were detected. For a full knockout, eight allelic mutations have to be induced, since the *ALS* gene is present as two highly similar gene copies (*ALS1* and *ALS2* on chromosome 3 and 7, respectively). Mutations were, however, detected in both genes and in different alleles, indicating that multiallelic mutagenesis is technically possible. In this case, *ALS* being an essential gene for biosynthesis of branched-chain amino acids, complete loss of function is not an option. Instead, the herbicide resistance phenotype is conferred by specific amino acid substitutions, which produce a trait that inherits dominantly. *ALS* would therefore be an obvious target for gene targeting (GT) and Butler et al. (2016) used HDR and a DNA repair template harboring a modified *ALS* DNA sequence with point mutations to create an herbicide-tolerant ALS variant. Without selection, no GT events were detected in 150 transgenic events expressing TALENs and a DNA repair template with modified *ALS,* suggesting that more sensitive methods were needed to recover events with modified *ALS*. Therefore, a DNA repair template was generated that contained a translational fused *NptII* coding sequence for kanamycin selection in addition to the modified *ALS* sequence. With this combined repair template, several gene targeted events could be selected on kanamycin-containing regeneration medium. Five of the nine evaluated events displayed reduced susceptibility to imidazolidinone herbicide in a greenhouse spraying assay, indicating a successful modification of *ALS* through gene targeting. Direct selection for an herbicide-tolerant phenotype on herbicide containing regeneration medium was not tested.

These early studies for targeted mutagenesis and gene targeting in tetraploid potato showed promising results and paved the way for modification of other interesting traits in tetraploid potato. Examples concern improvement of product quality, i.e. in keeping and processing quality of potatoes, in particular starch, which is used both in food and technical applications.

Potato starch is a mixture of amylose and amylopectin. Changing the ratio of these two components greatly alters the technical properties of the starch. The enzyme granule-bound starch synthase (GBSS) is responsible for the synthesis of amylose and in cultivated potato GBSS is encoded by a single locus (*GBSSI*), having four alleles. High amylopectin potatoes have been developed by random mutagenesis of dihaploid potato lines and selection of *GBSSI*-mutants (Hovenkamp-Hermelink et al. 1987) and subsequent regeneration and breeding steps to create tetraploid potato lines in which all 4 *GBSSI* alleles were mutated. Tetraploid potato lines with similar phenotypes were later also produced by antisense (Visser et al. 1991) and RNAi-mediated (Andersson et al. 2003) silencing of *GBSSI* expression. In a novel approach, Andersson et al. (2017, 2018) used protoplast technology and delivery of CRISPR/Cas9 targeting *GBSSI* as plasmid DNA and ribonucleoprotein (RNP), respectively, and demonstrated efficient multiallelic targeted mutagenesis of *GBSSI* in tetraploid potato. In the study using a DNA vector encoding the CRISPR/Cas machinery for delivery into the protoplasts, integration of the DNA vector was observed in 10% of the mutant lines, indicating that in the majority of mutant lines mutation induction was a result of transient expression of CRISPR/Cas (Andersson et al. 2017). To prevent any DNA integration, RNPs were also tested for so called ‘DNA-free’ delivery of the genome editing machinery. However, when using RNPs with in vitro transcriptionally produced guide RNA, more than 80% of the shoots with confirmed mutations had unintended inserts in the cut site, originating both from DNA template remnants left behind from the in vitro transcription of the gRNA, and from chromosomal potato DNA (Andersson et al. 2018). When using RNPs with synthetically produced guide RNA, no such unintended inserts were found. Both methods, employing transient expression from plasmid DNA or RNPs, resulted in 2–3% of the regenerated shoots having mutations in all four alleles resulting in a complete knockout of the GBSS enzyme function.

Other publications report similar successful genome editing studies in tetraploid potato, resulting in multiallelic targeted mutagenesis and non-transgenic mutant lines for several interesting traits. Clasen et al. (2016) used TALENs to target the vacuolar invertase gene (*VInv*), aiming at minimizing the accumulation of reducing sugars during cold storage of potato tubers, thus avoiding spots formation upon bruising, and acrylamide production through reaction with amino acids. TALENs were transiently expressed from plasmids in protoplasts and several plants with mutations in all *VInv* alleles and not containing TALEN DNA insertions in the genome were obtained. These lines showed undetectable levels of reducing sugars in their tubers. Gonzalez et al. (2020) used CRISPR/Cas9 RNPs for inducing mutations in the polyphenol oxidase 2 gene (*StPPO2*) in tetraploid potato and obtained as many as 24% of edited lines carrying mutations in all four alleles. This knockout led to a reduction of PPO activity in the tubers and a considerable reduction of enzymatic browning after cutting. Using *Agrobacterium*-mediated transient expression of TALENs in internodes of in vitro potato plantlets, and after subsequent shoot regeneration, Yasumoto et al. (2020) obtained mutant plants without integration of the TALENs transgenes. The TALENs targeted the sterol side chain reductase 2 gene (*SSR2*), which is involved in biosynthesis of steroidal glycoalkaloids that are toxic to consumers. Regenerated lines with all four *SSR2* alleles mutated showed reduced levels of α-solanine and α-chaconine in leaves of in vitro plants. Accumulation of steroidal glycoalkaloid in tubers was not studied.

These studies all yielded multiallelic mutations in a diversity of interesting genes, while unwanted integrations could be largely avoided. The improved plant lines without an SSN constructs in their genome demonstrate that genome editing is a robust technique for targeted mutagenesis in potato research and breeding.

In addition to targeted mutagenesis, precise editing using CRISPR/Cas9 base editors and prime editors has also been tested in potato. For precise editing of the catalytic motifs of GBSSI in tetraploid potato, a cytidine base editor, which directs a C-to-T base conversion at the target site, was explored by Veillet et al. (2019). They efficiently and precisely induced DNA substitutions in the KTGGL-encoding sequence of *GBSSI*, which is an essential domain for GBSS function (Nazarian-Firouzabadi and Visser 2017). The substitutions led to discrete variation in the amino acid sequence and generated a loss-of-function allele. Some mutant lines had mutations in all four alleles. They expanded this research by designing cytidine base editors with different PAM requirements, thereby broadening the set of potential target sites (Veillet et al. 2020a, 2020b). In their latest work they showed a proof of concept of application of a CRISPR/Cas9-mediated prime editor, which allows targeted ‘rewriting’ of short stretches genomic DNA sequences, in potato. Preliminary results show the expected substitution of three nucleotides at the *StALS1* target locus, which would provide herbicide tolerance (Veillet et al. 2020c).

## Deleting disease susceptibility factors and gluten genes and introducing traits facilitating introgression and hybrid breeding in hexaploid bread wheat

Bread wheat (*Triticum aestivum*) is the third cereal staple crop after maize and rice in terms of worldwide production. Bread wheat is an allohexaploid wheat species with a large ABD genome. Genome editing started early in bread wheat, with the first results on resistance to the powdery mildew *Blumeria graminis* f.sp. *tritici* (*Bgt*) (Wang et al. 2014). For obtaining resistance, the *MLO* gene was targeted, which represents a classical susceptibility gene (Pavan et al. 2010), i.e. suppressing its expression by e.g. knockout mutations will lead to disease resistance. In barley, an *mlo* mutation has shown durable effectiveness despite being used in commercial cultivation since the late 1970s (Kusch and Panstruga 2017). Wheat has three homoeologous gene copies, *TaMLO-A1*, *TaMLO-B1* and *TaMLO-D1*. Wang et al. (2014) used TALENs targeted to a conserved region in exon 2 of the *MLO* gene copies. The TALENs construct was co-transformed with a selectable marker gene, *bar*, into embryos using particle bombardment, and 3.4–6.0% of the regenerated transgenic plants (T0) showed mutations in *MLO* genes. Using primers specific for the A1, B1 and D1 genes, mutations were found in all copies, and it proved possible to obtain complete *tamlo-aabbdd* mutants in two generations of selfing. These mutant lines were also free of TALENs constructs, which was segregated out during selfing. Seedling leaves tested with *Bgt* conidiospores showed significant resistance in the *tamlo-aabbdd* (triple) mutant. No significant resistance was found for double mutants, demonstrating that all gene copies play a role in mildew infection. Resistance was effective for several *Bgt* races, thus showing that broad-spectrum resistance could be achieved. In comparison, using random EMS mutagenesis, Acevedo-Garcia et al. (2017) were also able to obtain *TaMLO* mutants with a TILLING approach. Selected mutations could also be stacked into a triple mutant *tamlo-aabbdd*, but this took six generations and 30 months starting with propagating the M2 for variety selection, and still unwanted mutations from mutagenesis treatment needed to be removed by backcrossing. Unlike the TALENs triple mutant of Wang et al. (2014), this triple mutant was partially resistant to *Bgt*, so the effect was also weaker. On the other hand, weaker *mlo* alleles may have the advantage of fewer pleiotropic negative effects on plant performance, such as early senescence, which were observed in the TALENs mutants. *TaMLO* was also used as target for a proof of concept of gene targeting using an HDR approach in protoplasts with a DNA-repair template containing a promotor-less blue fluorescent protein (*bfp*) coding sequence (Gil-Humanes et al. 2017). Correct gene targeting would result in *bfp* expression, which was observed at a frequency of up to 6.4% when using viral replicons for a high copy number delivery of repair template. Targeted integration of *bfp* was only observed into the *MLO* homoeoallele on chromosome D. The lower similarity between the homology arms of *bfp* DNA repair template and the A and B homoeoalleles (83 and 80.4%, respectively) might explain why GT of the *bfp* occurred preferentially at the D homoeoallele (which was 100% identitical).

Another interesting and important application of genome editing in wheat is aimed at the production of wheat that is safe for celiac patients, who do not tolerate immunogenic gluten proteins in their diet. Gluten quality is a rather complex trait. Bread wheat contains two groups of gluten proteins, glutenins and gliadins, which provide elasticity and viscosity to bread dough and are thus essential for good baking quality. Both glutenins and gliadins are comprised of protein groups (HMW and LMW glutenins and α-, γ-, and ω-gliadins, respectively) which are all encoded by multigene families. For example, in the bread wheat variety Chinese Spring, 29 α-gliadins, 18 γ-gliadins, 10 ω-gliadins, 6 HMW-, and 16 LMW-glutenins have been annotated (Clavijo et al. 2017). Most gluten proteins have immunogenic epitopes that trigger celiac disease. Different epitopes may be present, in varying number, resulting in variation in immunoreactivity across the gluten proteins. For α-gliadins also some variants that are free of immunogenic epitopes have been identified (Van Herpen et al. 2006; Salentijn et al. 2013; Schaart et al. 2021). So far, no traditional breeding strategies have been developed to produce ‘celiac-safe’ bread wheat. Targeted mutagenesis technology may however provide a route to obtain bread wheat containing safe or safer gluten proteins. In two studies, gliadin genes were targeted using CRISPR/Cas9 (Sanchez-Leon et al. 2018; Jouanin et al. 2019). Sanchez-Leon et al. (2018) targeted α-gliadin genes and observed in one line that, out of the 45 gliadin genes that were identified in the wild type, up to 35 genes were mutated and the immunoreactivity was reduced by 85% when compared to wheat from wildtype plants. Also, transgene-free wheat lines were obtained by segregating out the CRISPR/Cas9 construct by selfing. Following a similar genome editing approach, using guide RNAs targeting conserved domains in α-gliadin and γ-gliadin gene families simultaneously, mutant lines with a significant reduction in both α- gliadin and γ-gliadins were obtained (Jouanin et al. 2019). Because α-gliadin genes are located in tandem arrays, genome editing targeting conserved regions within these genes simultaneously may result in partial or complete deletion of neighboring gliadin genes copies, and evidence for that effect was also obtained (Jouanin et al. 2020a). The low-gluten wheat lines obtained in these studies provide a basis for subsequent editing studies to further reduce the epitope load in these wheat lines and to ultimately generate hypoimmunogenic-gluten wheat varieties (Jouanin et al. 2020b).

Several other genome editing studies for the improvement of various traits including grain and kernel weight and storability in wheat have been described and reviewed by Dayani et al (2019). Efficient multiplex genome editing targeting three different genes simultaneously in bread wheat was described recently (Li et al. 2020b).

Recombination between homoeologous chromosomes in allopolyploids such is needed for introgression of desirable traits, such as disease resistances, from more distantly related wild relatives with different ploidy levels. The Pairing homoeologous 1 (*Ph1*) locus allows only homologous chromosomes to pair and recombine during meiosis, and this causes the diploid inheritance of hexaploid wheat. This pairing behaviour prevents the multivalent formation that may occur in polyploid meiosis, and consequently avoids aneuploidy in the progeny. Regulatory gene(s) at the *Ph1* locus are responsible for homoeologous chromosome pairing control (HEPC) and targeting these genes could be a means to achieve exchange of useful genes between homoeologous chromosomes of the crop and wild relatives. However, after the intended exchange of genomic parts, the Ph1 function will need to be restored to prevent unwanted further chromosomal rearrangements in the new lines. *Ph1* is located on chromosome arm 5BL; the homoeologous loci on the A and D genomes are largely inactive. An effective loss-of-function mutant, *Ph1b*, is used in introgression and mapping studies with wild relatives. It was generated through a large deletion caused by X-ray irradiation mutagenesis. The *Ph1b* deletion was delimited to 60 MB (Gyawali et al. 2019) and other studies have brought it down to 2.4 MB using single-break chromosome deletion lines. As far as is presently known, multiple genes may be involved (see recent overview in Svačina et al. 2020). For two genes there are reports on their involvement in homoeologous recombination. Rawale et al. (2019) reported additional evidence on the candidate gene *C-TdPh1-B,* that was originally identified through bioinformatics on a syntenic rice region and mapped in wheat in the 2.4 MB region, based VIGS (virus-induced gene silencing) of selected genes in the region. Notably one of three splice variants, *C-TdPh1-B*^*alt1*^, was associated with homoeologous chromosome pairing control, based on a comparison with tetraploid *Triticum turgidum* ssp. *dicoccoides* lines differing in HEPC. This variant was expressed at much higher levels at early meiosis (chromosome pairing stage). However, Rey et al. (2018) reported that also *ZIP4* could be involved as indicated by TILLING mutants showing higher homoeologous recombination with wild relatives and by observations on a *Tazip4-B2* CRISPR wheat mutant hybrid with the wild relative *Aegilops variabilis*. Thus, it is not yet clear to what extent the Ph1 mechanism can be manipulated into an efficient introgression method in wheat, but it can be expected that CRISPR/Cas will be used to generate mutants in the relevant genes in this genomic region for the functional studies needed.

Recently, studies have been published on traits that may be of direct interest to wheat breeding programs aiming at creating hybrid wheat lines. Yield improvement in wheat is lagging behind cereals such as maize and hybrid breeding could give wheat breeding a boost (Longin et al. 2012; Whitford et al. 2013). To produce parental lines for hybrid breeding, the ability to create (doubled) haploid lines shortens the time required to produce such parental lines, which are traditionally produced by repeated selfing. For the generation of haploid-inducing lines in wheat the *MATRILINEAL* (*TaMTL*) gene was targeted by Liu et al. (2020). This gene encodes a pollen-specific phospholipase and was proven to trigger haploid induction in maize after mutagenesis (Kelliher et al. 2017; Liu et al. 2017). A double-knockout mutation of the *TaMTL* homeologs on the A and D genome (*TaMTL-4A* and *TaMTL-4D*) resulted in haploid induction at a frequency of 10%, and triple-knockout of *TaMTL-4A*, *TaMTL-4B*, and *TaMTL-4D* resulted in haploid induction up to a frequency of 31.6%.

Pure hybrid seed production requires maternal lines from which no viable male gametes are introduced. This can be achieved by artificial emasculation, which is labor-intensive and time-consuming and therefore an unsuitable method for large-scale hybrid seed production in wheat. In maize and rice male-sterile lines have been developed, which make emasculation unnecessary. in combination with lines with fertility restorer genes, the male-sterile lines provide an effective system facilitating hybrid breeding. Although male-sterile germplasm has been discovered in wheat more than 40 years ago (Deng and Gao 1982), effective hybrid breeding was hampered by the lack of a fertility restorer line. Whereas the male-sterile systems in maize and rice are based on recessive genes, the wheat male-sterile line contains a dominant gene, *Ms2*, which controls complete male-sterility. A few years ago, the *Ms2* gene was identified as a pseudogene, which was reactivated due to the insertion of a retrotransposon in the promoter region of the *ms2* gene on the D-genome, while the other two *ms2* alleles on the A- and B-genomes are still inactive pseudogenes (Ni et al. 2017; Xia et al. 2017). Very recently Tang et al. (2020) edited *Ms2* for restoring the male-fertility in sterile wheat lines using CRISPR/Cas9, and they recovered wheat lines in which fertility was completely recovered, rendering an effective *MS2*-based hybrid breeding system for wheat.

Other genome editing studies aimed at generating novel male-sterile mutant lines. By targeting all homeologs of the *TaMs45* gene (Singh et al. 2018), the *TaMs1* gene (Okada et al. 2019) or the *TaNP1* gene (Li et al. 2020a) in wheat, mutant lines were rapidly obtained with complete male-sterility. Introgression of such three-genome mutations into different genetic backgrounds through backcrossing would be laborious and challenging, but using genome editing male-sterility can now easily be introduced in any important wheat variety or breeding line. The successful engineering of both the haploid-inducing and the male-sterility traits in wheat was based on a choice of target genes directly derived from results obtained in rice and maize. This successful choice of genes demonstrates the strength of genome editing technology to directly translate knowledge on traits from one species to another if a genome sequence is available. The developments described above may speed up the innovation of hybrid wheat breeding with the potential to bring about higher-yielding, better-adapted wheat lines.

## Changing fruit color in octoploid strawberry

Strawberry (*Fragaria x ananassa*) and sugarcane (*Saccharum* spp. hybrids) are examples of crops with an exceptionally large number of homologous chromosome sets. Cultivated strawberry has a highly heterozygous octoploid genome, which makes generating improved varieties through traditional breeding a challenging practice. Therefore, new breeding techniques, including genome editing, may facilitate breeding efforts in strawberry. Recently genome editing using CRISPR/Cas9 in octoploid strawberry has been described by different groups. Targeting *PDS* in stably transformed strawberry lines resulted in plants with a clear albino phenotype at a high frequency, suggesting that genome editing is not limited by genome complexity (Wilson et al. 2019). Indeed, Illumina MiSeq sequencing of *PDS* amplicons showed that 60–80% of the albino shoots had targeted mutations in all *pds* alleles. However, some albino shoots still contained wildtype *PDS* alleles and sequence data showed that these albino shoots had up to 31% wild-type reads. Interestingly, one green mutant shoot had only 11% wild-type reads. These results suggest that in octoploid strawberry just a single *PDS* allele may suffice for producing the enzyme for normal functioning of the plant and that the other wildtype *PDS* alleles are non-functional. To characterize the function of the MADS box transcription factor *FaTM6* in octoploid strawberry flower development, Martin-Pizarro et al. (2019) performed targeted mutagenesis with CRISPR/Cas9. The mutant lines they obtained showed the expected phenotypes with modified anthers, even though none of these lines had mutations in all *FaTM6* alleles, confirming the notion that some of those are non-functional alleles. Gao et al. (2020) observed the same phenomenon when targeting the *Reduced Anthocyanins in Petioles* (*RAP*) gene. They obtained altered fruit coloration in cultivated strawberry plants in which only six of the eight *RAP* alleles were knocked out. Loss of functional alleles as well as functional diversification seems to be a common phenomenon in the evolution of (allo)polyploids and is well-documented by Wendel et al. (2018). Therefore, the common presence of non-function alleles implies that when using mutagenesis in polyploids, it will not always be required to modify the full allelic gene set to produce a mutant phenotype.

## Changing lignin content in decaploid sugarcane

Sugarcane has one of the most complex genomes among crop species due to the extreme level of polyploidy with 10 to 13 homologous chromosome sets (including aneuploid sets). In an effort to perform targeted mutation in sugarcane, TALENs were employed to induce mutations in a highly conserved region of the caffeic acid O-methyltransferase (*COMT*) gene (Jung and Altpeter 2016). COMT is involved in the biosynthesis of lignin and suppression of lignin formation will improve saccharification and consequently facilitate the production of biofuel from lignocellulosic material. Pyrosequencing of the COMT gene revealed that in 74% of the transgenic lines that contained TALENs, 8–99% of the wild type *COMT* were converted to mutant *COMT*. Events with a mutation frequency of 99% displayed the expected phenotype of reduction of the lignin content of 29–32%, compared to non-transgenic control lines. Later it was reported that, based on Sanger sequencing of cloned *COMT* amplicons, as many as 107 of the 109 unique *COMT* copies/alleles had been edited in one of these lines (Kannan et al. 2018). This research demonstrates the incredible effectivity of TALENs for targeted mutagenesis of a very large number of alleles/copies for improvement in crops with complex genomes.

## Prospects

The cases described in this review demonstrate that genome editing is a very effective and robust tool tor targeted mutagenesis in auto- and allopolyploids, even if they have a high level of ploidy, such as sugarcane. Genome editing is therefore a promising method for introducing functional mutations for direct improvement of polyploid crops.

One class of application is the modification of one or a few traits in elite crop varieties that are propagated vegetatively. In most cases this concerns outcrossing species, where the optimal combination of characters of the elite variety is lost upon crossing as the traits will segregate in the progeny (Schaart et al. 2015). Successful varieties are often cultivated for a long time. Examples are potato, where a factory may be fine-tuned to process one particular potato variety, and fruits such as apple, where supermarkets expect a large volume of a variety that consumers already know and appreciate. This relative inertness makes it attractive to be able to ‘upgrade’ varieties or breeding lines with proven quality.

For genome editing in such vegetatively propagated, highly heterozygous crops it is imperative to have methods that avoid integration of the DNA containing the SSN in the genome, as backcrossing for removing a construct integrated into the genome is not an option. Using protoplast technology, transgene-free lines with multi-allelic mutations have effectively been produced in, e.g., potato (Andersson et al. 2017). However, in wheat, as in several other species, it is not possible to regenerate plants from protoplasts. In addition, regeneration of plants from protoplasts may lead to genome instability resulting in aneuploidy or structural chromosomal changes, as has been reported for potato (Ramulu et al. 1986; Fossi et al. 2019).

Yasumoto et al. (2020) obtained mutant potato plants without integration of the TALENs transgenes by *Agrobacterium*-mediated transient expression in plant tissue. In transformation experiments usually selective regeneration media are used for the recovery of transgenic lines, but by omitting selection Yasumoto et al. (2020) obtained shoots with mutations but without integrated transgenes. For wheat, alternative systems have been reported, such as transient expression in calli (Zhang et al. 2016) or RNP delivery into immature embryos (Liang et al. 2017). Similar approaches could also be applied to other clonally propagated polyploids such as apple or strawberry. Ma et al. (2020) developed an interesting approach for genome editing without the need for transgene integration by making use of viral delivery of CRISPR-Cas9. Viruses are known for their limited packaging capacity, which impedes the delivery of the large CRISPR/Cas9 cassette (> 4.5 kb), but this technical barrier was overcome by using Rabdoviruses. Ma et al. (2020) engineered the Sonchus yellow net rhabdovirus (SYNV) for *in planta* delivery of CRISPR/Cas9 reagents and demonstrated mutagenesis and chromosome deletions at high frequency in infected allotetraploid tobacco. Because plant rhabdoviruses do not invade meristematic or germline cells, mutagenesis was restricted to somatic tissue and progeny of engineered SYNV-infected plants did not show any gene edits. Using tissue culture technology, heritable, virus-free shoots could be regenerated from infected somatic tissue. The authors envision that this strategy is potentially applicable to rhabdoviruses that infect crop species and so would also have potential for the generation of gene-edited non-transgenic polyploid crops.

As is already clear from the studies described in this review and elsewhere, one very important application of genome editing in polyploid crops concerns disease resistances. Modifying R genes, for instance to repair dysfunctional genes, is one approach. A second type of resistance is generated through the use of susceptibility genes (Wang et al. 2014; Schaart et al. 2015). As the resistance is based on the absence of a functional gene, it generally behaves as a recessive trait. This is more challenging in a polyploid crop with multiple (homoeologous) gene copies, as in the breeding process all alleles should be replaced by non-functional alleles. In a breeding program for an outcrossing crop such as tetraploid rose, combining existing non-functional S alleles may lead to a reduction of heterozygosity, and this may affect the quality of the prospective varieties. Genome editing could be used here to directly modify elite cultivars, but also to increase the number and frequency of non-functional alleles in a breeding program, so that homozygosity at S gene loci may be achieved without affecting the diversity in the remainder of the genome. In that way, genome editing contributes to a more sustainable agriculture with less chemical inputs, without a trade-off in the form of decreased heterozygosity and/or diversity.

Scientifically interesting was the detection of the occurrence of natural non-functional alleles in e.g. the studies described for strawberry. For polyploids it is known that genes may have undergone drastic changes in structure and function as a result of genetic and epigenetic changes during polyploidization (Osborn et al. 2003). On an evolutionary scale, a polyploidization (WGD) event is usually followed by a diploidization process, involving gradual loss or conversion of duplicate genes at an exponential rate according to analysis of 141 genomes by Qiao et al. (2019). Alternatively, duplicate genes may gain a new functionality, which is possible as the gene redundancy leaves more opportunities for maintenance of deleterious alleles without affecting the fitness of individuals. There can be a bias towards one of the genomes, but this may vary with specific allopolyploids, and may be related to epigenetic effects with the involvement of transposable elements that can be activated upon polyploidization (Wendel et al. 2018). Structural changes may already occur relatively quickly and more or less consistently. This could be studied with experimentally resynthesized polyploids, which may be compared to what is found in the existing polyploid, such as with hexaploid wheat. For the study of phenomena such as possible mechanisms behind biased gene loss, comparison of recently formed (neo)polyploids and resynthesized versions, a CRISPR/Cas9 system was developed for the *Tragopogon* species involved in neopolyploidization (Shan et al. 2018). When using genome editing in polyploids, phenotypic changes are compared with genotypic changes, and this will therefore also produce information on the occurrence of non-functional alleles for a range of traits in the germplasm of several crop species.

Insight in the number of alleles in a population is still scarce, and overviews of how many of them are non-functional is rare. It has been difficult to assess the genomic changes in polyploids due to complexities with whole genome sequencing, as particularly structural variations can easily be overlooked. With the development of long-read technologies and refined algorithms, this has improved. Genetic variation is incorporated into pan-genomes, such as e.g. for *Brassica napus* (Song et al. 2020). For the hexaploid but also particularly large genome of wheat, this has also been started by mapping to a refined reference genome (Montenegro et al. 2017) or limiting the analysis to the exome (Pont et al. 2019). For the octoploid strawberry and the decaploid and higher sugarcane, the first genome sequences have been reported (Edger et al. 2019; Souza et al. 2019). For breeding for specific traits, this improved sequencing is helpful to precisely identify all variant alleles, including the non-functional pseudogenes. The high specificity of CRISPR/Cas in targeting and the possibility of multiplexing guide RNAs, provides flexibility in targeting. So, one may target just a single specific allele based on a unique feature while leaving all other alleles untargeted or target all allelic variants of a gene in case of a high level of heterozygosity, using a conserved region.

The possibility to generate allelic series of mutants enables studying allele dosage effects. We speculate that dosage effects could be a widespread phenomenon in polyploid crops, which could be exploited to fine-tune particular trait phenotypes in breeding. We base this on results from GWAS studies in polyploids, which indicate dosage effects for the number of alleles present at particular loci, for instance for petal number in tetraploid rose (Hibrand Saint-Oyant et al. 2018) and for several yield-related traits in bread wheat (Li et al. 2019). An interesting example was described for oilseed rape (OSR) by Song et al. (2020): Vernalization requirements were shown to be related to dosage effects from structural variations in three *Flowering Locus C* (*FLC*) genes, with *BnaA10.FLC* being the master regulator. The slight vernalization requirement of Spring OSR was likely due to the *BnaA10.FLC* copy having exon 1 with a LINE type transposable element insertion, leading to a loss-of-function. In Winter OSR the high vernalization requirement was related to the promoter of the *BnaA10.FLC* copy, which had a MITE type transposable element insertion leading to higher expression. Semi-Winter OSR was intermediate with an insertion of a hAT type transposable element in the *BnaA10.FLC* promoter.

Most of the genome editing examples described in this review are aimed at improving crop quality or cultivation characteristics by reducing undesirable traits through knockout mutations. Specific applications of genome editing technology can also directly facilitate the breeding process of crops with complex genomes, for example the application of genome editing in wheat to introduce male sterility and haploid inducing capacity, both supporting hybrid cultivar breeding. It may also be used to domesticate candidate crops (Fernie & Yan 2019; Lemmon et al. 2018; Van de Wiel et al. 2017). Several other possibilities exist. HDR-mediated targeted allele replacement makes it possible to replace undesirable alleles with more beneficial ones. Targeted recombination can be used to induce desired chromosomal recombination events (Rönspies et al. 2021). Reversing chromosomal inversions, thereby resolving recombination suppression, facilitates e.g. the removal of undesirable traits from regions introgressed from wild relatives (Schmidt et al. 2020). Shortening the juvenile phase will speed up breeding, and this may be achieved e.g. by targeting the *FLC* gene, as was described or *Brassica rapa* (Jeong et al. 2019). Many of these applications of gene editing may be expected to have an important contribution to the future breeding of improved polyploid crops.
